# *DDX18* Facilitates the Tumorigenesis of Lung Adenocarcinoma by Promoting Cell Cycle Progression through the Upregulation of CDK4

**DOI:** 10.3390/ijms25094953

**Published:** 2024-05-01

**Authors:** Bingbing Feng, Xinying Wang, Ding Qiu, Haiyang Sun, Jianping Deng, Ying Tan, Kaile Ji, Shaoting Xu, Shuishen Zhang, Ce Tang

**Affiliations:** 1 Department of Thoracic Surgery, The First Affiliated Hospital, Sun Yat-sen University, Guangzhou 510080, China; 2 Institute of Precision Medicine, The First Affiliated Hospital, Sun Yat-sen University, No. 58, Zhong Shan Er Lu, Guangzhou 510080, China; 3 Animal Experiment Center, The First Affiliated Hospital, Sun Yat-sen University, Guangzhou 510080, China

**Keywords:** *DDX18*, lung adenocarcinoma, cell cycle progression, CDK4, c-MYC

## Abstract

Lung adenocarcinoma (LUAD) is the most prevalent and aggressive subtype of lung cancer, exhibiting a dismal prognosis with a five-year survival rate below 5%. DEAD-box RNA helicase 18 (*DDX18*, gene symbol *DDX18*), a crucial regulator of RNA metabolism, has been implicated in various cellular processes, including cell cycle control and tumorigenesis. However, its role in LUAD pathogenesis remains elusive. This study demonstrates the significant upregulation of *DDX18* in LUAD tissues and its association with poor patient survival (from public databases). Functional in vivo and in vitro assays revealed that *DDX18* knockdown potently suppresses LUAD progression. RNA sequencing and chromatin immunoprecipitation experiments identified cyclin-dependent kinase 4 (CDK4), a cell cycle regulator, as a direct transcriptional target of *DDX18*. Notably, *DDX18* depletion induced G1 cell cycle arrest, while its overexpression promoted cell cycle progression even in normal lung cells. Interestingly, while the oncogenic protein c-Myc bound to the *DDX18* promoter, it did not influence its expression. Collectively, these findings establish *DDX18* as a potential oncogene in LUAD, functioning through the CDK4-mediated cell cycle pathway. *DDX18* may represent a promising therapeutic target for LUAD intervention.

## 1. Introduction

Lung cancer remains a significant global health burden. In 2022, an estimated 20 million new cancer diagnoses were reported, alongside 9.7 million cancer-related deaths [1]. Lung cancer emerged as the most prevalent malignancy, accounting for nearly 2.5 million new cases (12.4% of all cancers). Furthermore, lung cancer held the dubious distinction of being the leading cause of cancer mortality, responsible for an estimated 1.8 million deaths (18.7% of all cancer deaths) [1]. Lung cancers can be classified into small cell lung carcinoma and non-small cell lung carcinoma (NSCLC), with the latter accounting for approximately 85% of all cases [2]. Recent breakthroughs in pathway understanding, detection technologies, and targeted therapies have revolutionized treatment for lung adenocarcinoma [3]. Identification of actionable mutations, such as those in EGFR, PI3K/AKT/mTOR, RAS–MAPK, and NTRK/ROS1 pathways, has led to the development of highly effective drugs [4]. These include EGFR inhibitors (erlotinib, gefitinib) and targeted therapies for other pathways (everolimus, entrectinib), some of which now surpass chemotherapy as first-line treatment [4]. However, survival in NSCLC patients remains poor due to early metastases and the development of targeted therapy resistance [5].

**Figure 1:**
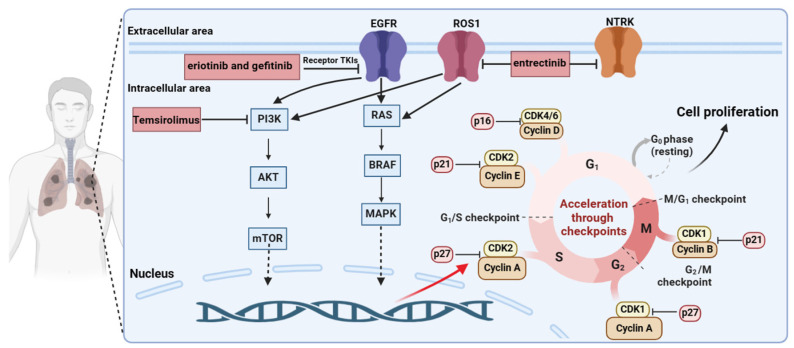
Mechanisms of lung cancer development. Dysregulation of signaling pathways, including EGFR, PI3K/AKT/mTOR, RAS-MAPK, NTRK/ROS1, and cell cycling has been implicated in lung cancer development and progression. Targeted therapies capitalizing on these pathways have emerged as a cornerstone of treatment. Inhibitors such as entrectinib, gefitinib, and temsirolimus target specific oncogenic drivers like EGFR, ROS1, and RAS, demonstrating efficacy in controlling lung cancer progression.

Tightly regulated cell cycle progression is essential for maintaining cellular homeostasis. The four distinct stages (G1, S, G2, and M) are governed by the coordinated activity of cyclin-dependent kinases (CDKs) and cyclin proteins [6,7,8,9]. Cell cycle-specific transcriptional control and protein degradation mechanisms further ensure the precise temporal regulation of these proteins [10]. During the G1 phase, in response to mitotic signals, D-type cyclins (D1, D2, and D3) bind and activate CDK4/6, leading to retinoblastoma protein (RB) phosphorylation [11,12]. This phosphorylation disrupts the E2F–RB complex, releasing the E2F transcription factor and promoting the expression of E-type cyclins (E1, E2). Subsequently, cyclin E–CDK2 complex formation drives the G1/S transition in concert with cyclin D-CDK4/6 [11,13,14,15]. The S/G2 transition is facilitated by cyclin A-CDK2-mediated phosphorylation of DNA replication machinery components [16,17]. Finally, B-type cyclins and CDK1 orchestrate the G2/M phase transition [7,18,19]. Disruptions in cell cycle control mechanisms are a hallmark of cancer, including lung adenocarcinoma. Targeting these pathways offers a promising therapeutic strategy [8,11,12,17] (Figure 1). Besides cell biological factors, external mechanical stimulation, such as local nanomechanical forces prevalent within the soft matter environment [20] of the tumor extracellular matrix, is regulated by matrix stiffness and modulates PD-L1 expression through the activation of the Yes-associated proteins to contribute to tumor cell proliferation [21].

RNA helicases are a diverse group of enzymes essential for RNA metabolism, influencing various cellular processes such as translation, splicing, and RNA decay [22]. Notably, the DEAD-box RNA helicase (DDX) family represents the largest class of RNA helicases, with over 40 members, and plays roles in various cellular processes, mainly involving RNA but sometimes other nucleic acids [23]. Growing evidence implicates their involvement in tumorigenesis [24,25]. For example, MTR4 selectively cleaves mRNAs for glycolytic enzymes PKM2 and GLUT1, thereby regulating their expression and contributing to tumor development [26]. In breast cancer, DDX21 promotes tumorigenesis through distinct mechanisms, including the activation of the AP-1 signaling pathway and the modulation of rRNA processing [27]. Likewise, DDX17 functions as a transcriptional regulator, driving the binding of the nuclear Y-box binding protein 1 (YB1) to the epidermal growth factor receptor (*EGFR*) gene and promoting its transcription, ultimately facilitating liver cancer metastasis [28]. These findings collectively highlight the DDX family as a promising target for cancer therapy.

*DDX18*, a member of DEAD-box RNA helicases, is also involved in a broad range of functions associated with RNA. Similar to other DDX family members such as DDX5 and DDX10, *DDX18* also possesses a modular architecture with N- and C-terminal domains flanking a conserved helicase core composed of two RecA-like domains [29]. Emerging evidence suggests a multifaceted role for *DDX18* in cancer progression. Studies have demonstrated its upregulation and association with poor patient survival in pancreatic ductal adenocarcinoma (PDA) [30]. Mechanistically, *DDX18* promotes tumor immune escape in PDA by transcriptionally activating STAT1 expression [30]. Conversely, in gastric cancer, *DDX18* promotes tumorigenesis through a distinct pathway. Here, it facilitates the maturation of microRNA-21 via the activation of the AKT signaling pathway [31]. Interestingly, a recent report highlights a protective role for *DDX18*. It prevents R-loop-induced DNA damage and genome instability through its interaction with PARP-1 [32]. These findings collectively suggest that *DDX18* exerts context-dependent effects on cancer development.

To investigate the role of *DDX18* in lung adenocarcinoma (LUAD) development, we employed a multifaceted approach. Initially, gene chip technology was utilized to identify *DDX18* upregulation in LUAD tissues. Subsequently, functional assays were conducted to evaluate the impact of *DDX18* on LUAD cell behavior. Finally, transcriptome sequencing and in vitro experiments were employed to elucidate the underlying regulatory mechanisms by which *DDX18* contributes to LUAD progression.

## 2. Results

### 2.1. DDX18 Is Upregulated in the LUAD Tissue and Is Required for LUAD Cells Proliferation

**Figure 2:**
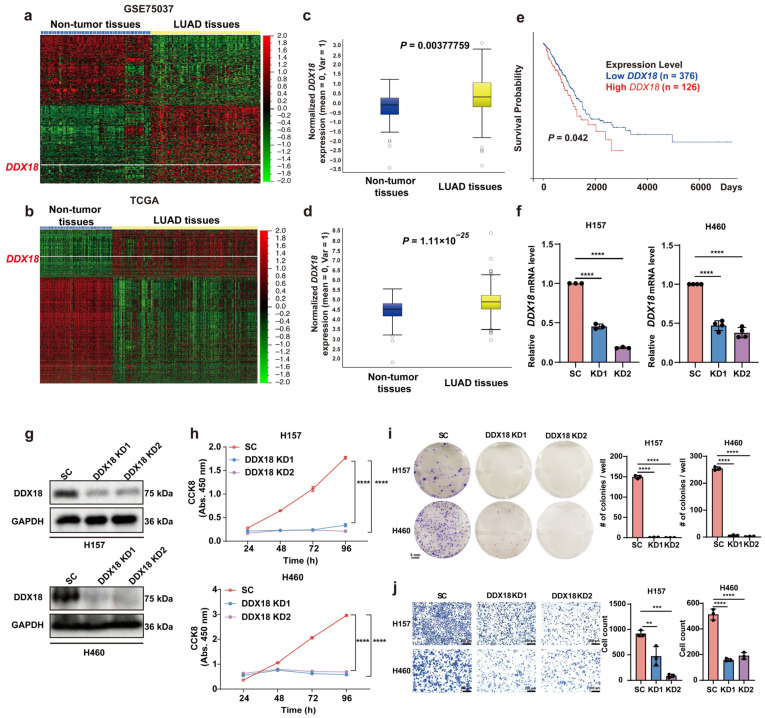
*DDX18* Upregulation in LUAD Tissues and its Functional Role in LUAD Cells. (**a**,**b**) Heatmaps depicting global mRNA expression profiles in GSE75037 [(**a**) non-tumor tissues (n = 83), LUAD tissues (n = 83)] and TCGA [(**b**) normal lung tissues (n = 287), LUAD tissues (n = 574)] datasets. *DDX18* expression is indicated by the white line. (**c**,**d**) Boxplots representing *DDX18* mRNA expression levels in non-tumor and LUAD tissues from GSE75037 (**c**) and TCGA (**d**) datasets. *p*-values are indicated. (**e**) Kaplan–Meier survival curve for overall survival of LUAD patients with high (n = 126) and low (n = 376) *DDX18* expression. Low *DDX18* expression correlates with improved overall survival (*p*-value indicated). (**f**) Relative *DDX18* mRNA levels in H157 (n = 3) and H460 (n = 4) cells following *DDX18* knockdown. Data represent mean ± SD. (**g**) Western blot analysis confirming *DDX18* knockdown efficiency in H157 and H460 cells. (**h**) *DDX18* knockdown significantly suppresses cell proliferation in H157 and H460 cells (n = 3, mean ± SD). (**i**) *DDX18* knockdown substantially reduces colony formation capacity in H157 and H460 cells (n = 3, mean ± SD). (**j**) *DDX18* knockdown inhibits the migration of H157 and H460 cells in vitro (n = 3, mean ± SD). ** *p* < 0.01; *** *p* < 0.001; **** *p* < 0.0001. LUAD, lung adenocarcinoma; SC, scramble control; KD, knockdown.

To evaluate the clinical significance of *DDX18* expression in LUAD, we analyzed publicly available datasets. *DDX18* mRNA levels were significantly higher in LUAD tissues compared to adjacent normal tissues in both the GSE75037 dataset (Figure 2a,c) and The Cancer Genome Atlas (TCGA) data (Figure 2b,d). Additionally, high *DDX18* expression correlated with poor prognosis in LUAD patients (Figure 2e).

To investigate the functional role of *DDX18* in LUAD cells, we generated *DDX18* knockdown H157 and H460 cell lines using lentiviral vectors expressing two distinct *DDX18*-specific shRNAs. Control cells were infected with a lentivirus containing a scramble shRNA. RT-PCR and Western blot analyses confirmed efficient *DDX18* knockdown in both cell lines (Figure 2f,g). Functional assays revealed that *DDX18* depletion significantly inhibited cell proliferation compared to the control (Figure 2h). Consistent with this finding, colony formation assays demonstrated a reduction in colony numbers in *DDX18* knockdown H460 and H157 cells compared to the control (Figure 2i). Furthermore, *DDX18* knockdown significantly impaired cell migration in both H460 and H157 cells (Figure 2j).

### 2.2. DDX18 Overexpression Promotes Tumor Cell Proliferation and Migration

**Figure 3:**
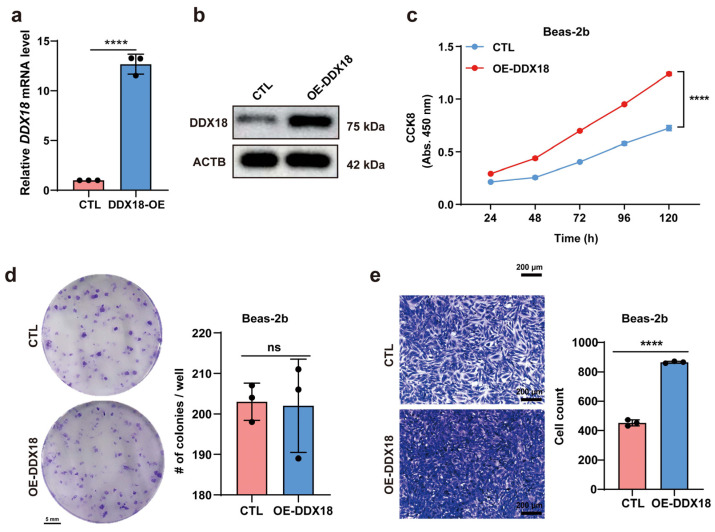
*DDX18* Overexpression Promotes Proliferation and Migration in Beas-2B Cells. (**a**) RT-PCR analysis confirming *DDX18* overexpression in Beas-2B cells. (**b**) Western blot analysis demonstrating *DDX18* overexpression efficiency in Beas-2B cells. (**c**) CCK8 assays measuring the proliferation of *DDX18*-overexpressing (OE-*DDX18*) and control Beas-2B cells (n = 3 independent experiments). Statistical differences were determined by two-way ANOVA followed by Bonferroni post-tests. (**d**) Colony formation assay evaluating the long-term effects of *DDX18* expression on clonogenic survival in Beas-2B cells. Cells were transfected with plasmids encoding *DDX18*, selected, replated, and assessed for colony formation. (**e**) Transfection with *DDX18* plasmids enhances Beas-2B cell migration in vitro. **** *p* < 0.0001; ns means non-significant. OE, overexpressed; CTL, control.

Building on the observation that *DDX18* silencing suppressed proliferation and migration of LUAD cells, suggesting its oncogenic role in LUAD, we hypothesized that *DDX18* overexpression might promote proliferation in non-malignant lung cells. Beas-2B cells were stably transduced with lentiviral vectors expressing *DDX18*. RT-PCR and Western blot analyses confirmed efficient *DDX18* overexpression at both mRNA and protein levels (Figure 3a,b). Notably, *DDX18* overexpression significantly enhanced Beas-2B cell proliferation as measured by the CCK8 assay (Figure 3c). However, colony formation assays revealed no significant difference in clonogenic survival between control and *DDX18*-overexpressing Beas-2B cells (Figure 3d). Conversely, *DDX18* overexpression demonstrably increased the migratory capacity of Beas-2B cells (Figure 3e).

### 2.3. DDX18 Knockdown Inhibits LUAD Tumorigenesis In Vivo

**Figure 4:**
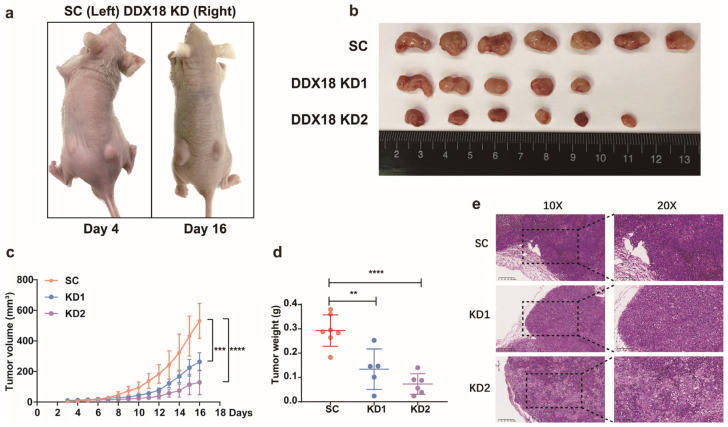
*DDX18* Knockdown Inhibits LUAD Tumor Growth In Vivo. (**a**) Representative images of tumor-bearing nude mice injected subcutaneously with H460 cells expressing either scramble shRNA (left flank) or *DDX18* shRNA (right flank) at 4 and 16 days post-injection. (**b**) Images of xenograft tumors formed by H460 cells with or without *DDX18* knockdown 16 days after injection. (**c**,**d**) Tumor volume (**c**) and weight (**d**) of xenografts derived from H460 cells expressing scramble shRNA (SC) or *DDX18* shRNA (KD1 and KD2) in nude mice. Data are presented as mean ± SD. (**e**) Representative hematoxylin and eosin (H&E) staining of tumor sections from mice injected with H460 cells expressing scramble shRNA (SC) or *DDX18* shRNA (KD1 and KD2). Scale bar, 200 μm. (n = 7 per SC group, n = 5 per KD1 group, n = 6 per KD2 group). ** *p* < 0.01; *** *p* < 0.001; **** *p* < 0.0001. SC, scramble control; KD, knockdown.

To evaluate the in vivo role of *DDX18* in LUAD tumorigenesis, H460 cells expressing either scramble shRNA (control) or *DDX18* shRNA were injected subcutaneously into the left and right flanks, respectively, of nude mice. Representative images of tumor-bearing mice were captured at 4 and 16 days post-injection (Figure 4a,b). Compared to control tumors, *DDX18* knockdown significantly suppressed LUAD tumor growth, as evidenced by reduced tumor volume (Figure 4c) and weight (Figure 4d). Hematoxylin and eosin (H&E) staining revealed a looser cell organization in *DDX18* knockdown tumor sections compared to controls (Figure 4e). These findings demonstrate that *DDX18* is essential for LUAD tumorigenesis.

### 2.4. Screening for Candidate Targets for *DDX18* through RNA-Seq and GO Enrichment Analysis

**Figure 5:**
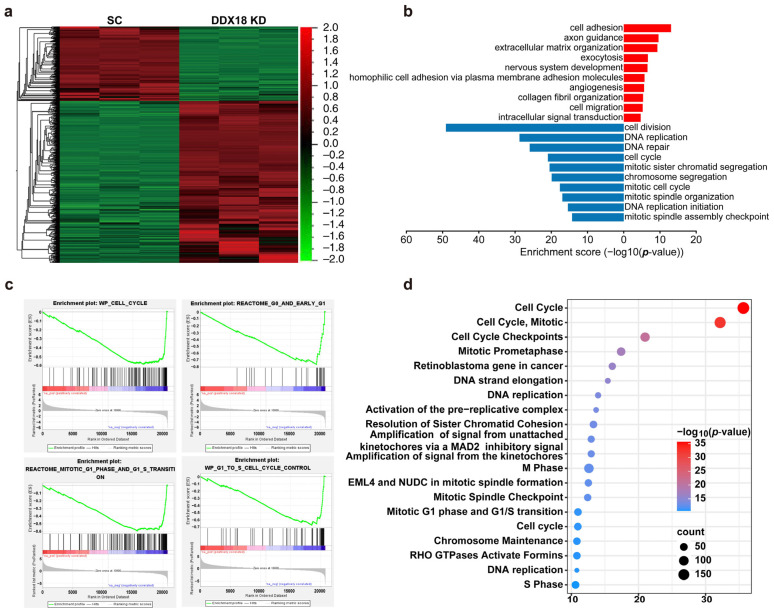
Identification of *DDX18* Regulatory Pathways using RNA-Seq and GO Enrichment Analysis. (**a**) Heatmap depicting differentially expressed genes (*p* < 0.05) between *DDX18* knockdown and scramble control H460 cells. (**b**) Top 10 enriched Gene Ontology (GO) pathways for upregulated and downregulated genes in *DDX18* knockdown H460 cells (*p* < 0.05). Average *p*-values are shown. (**c**) GSEA enrichment plots for cell cycle-associated gene sets in *DDX18* knockdown H460 cells. (**d**) Bubble plots illustrating enriched pathways in control and *DDX18* knockdown H460 cells. SC, scramble control; KD, knockdown.

RNA sequencing analysis was performed to comprehensively identify genes regulated by *DDX18* in H460 LUAD cells. Compared to scramble control cells, *DDX18* knockdown resulted in differential expression of over 2507 genes (fold change > 2.0) (Figure 5a). Functional enrichment analysis using KEGG pathways and Gene Ontology (GO) terms revealed a significant enrichment of differentially expressed genes (DEGs) in cell cycle-associated pathways. The top 10 enriched GO terms for upregulated and downregulated genes are presented in Figure 5b. Gene Set Enrichment Analysis (GSEA) further confirmed that the cell cycle pathway was significantly deregulated in *DDX18*-depleted LUAD cells (Figure 5c). Within the biological process (BP) category, GO enrichment analysis identified the most significant enrichments in categories related to “cell cycle,” “mitotic cell cycle,” and “cell cycle checkpoints” (Figure 5d). These findings suggest that *DDX18* plays a critical role in regulating cell cycle progression in LUAD cells.

### 2.5. DDX18 Knockdown Induces G1 Cell Cycle Arrest in LUAD Cells

**Figure 6:**
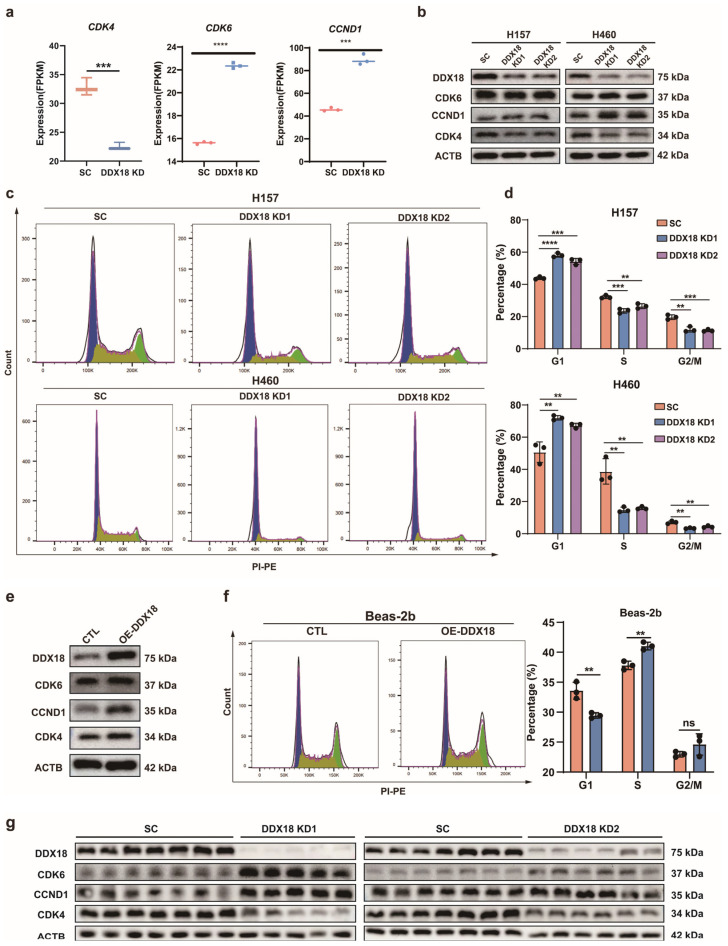
*DDX18* Knockdown Induces G1 Cell Cycle Arrest in LUAD Cells. (**a**) The mRNA levels of *CDK4*, *CDK6*, and *CCND1* in RNA-seq. (**b**) The protein levels of CDK4, CDK6, and CCND1 after knocking down *DDX18* in H157 and H460 cells. (**c**) Effect of *DDX18* knockdown on cell cycle distribution. H460 and H157 cells with or without *DDX18* knockdown were stained with propidium iodide for flow cytometric analysis. Histograms show the numbers of cells/channel (y-axis) vs. DNA content (x-axis). (**d**) Values indicate the percentages of cells in the corresponding phases of cell cycle. (**e**) The protein levels of CDK4, CDK6, and CCND1 after overexpression of *DDX18* in Beas-2b cells. (**f**) Effect of *DDX18* overexpression on cell cycle distribution. Beas-2b cells with or without *DDX18* overexpression stained with propidium iodide for flow cytometric analysis. Histograms show the numbers of cells/channel (y-axis) vs. DNA content (x-axis). (**g**) The protein levels of CDK4, CDK6, and CCND1 in subcutaneous tumors of nude mice. ** *p* < 0.01; *** *p* < 0.001; **** *p* < 0.0001; ns means non-significant. SC, scramble control; KD, knockdown; OE, overexpressed; CTL, control.

RNA-seq analysis identified CDK4, a key cell cycle regulator, as downregulated upon *DDX18* knockdown in H460 cells (Figure 6a). CDK4 deregulation is a well-established hallmark of various cancers. Interestingly, CDK6 and CCND1, both associated with G1-S phase transition, were upregulated in the absence of *DDX18* (Figure 6b). This suggests that *DDX18* might regulate cell proliferation by specifically targeting CDK4, a critical G1 phase checkpoint protein. Western blot analysis confirmed that protein levels of CDK4 mirrored the RNA-seq data, whereas CDK6 and CCND1 were upregulated following *DDX18* knockdown (Figure 6b). Flow cytometry analysis revealed a significant G1 arrest in H460 and H157 cells with *DDX18* knockdown, as evidenced by the increased G1 cell population (Figure 6c,d). Conversely, *DDX18* overexpression in Beas-2B cells led to increased protein levels of CDK4, CDK6, and CCND1 (Figure 6e) and altered cell cycle distribution with changes observed across G1, S, and G2/M phases (Figure 6f). Notably, protein levels of CDK4, CDK6, and CCND1 in nude mice xenografts mirrored those observed in cell lines (Figure 6g). These findings collectively suggest that *DDX18* regulates cell cycle progression, potentially through CDK4 modulation.

### 2.6. DDX18 Knockdown Induces Apoptosis in LUAD Cells

**Figure 7:**
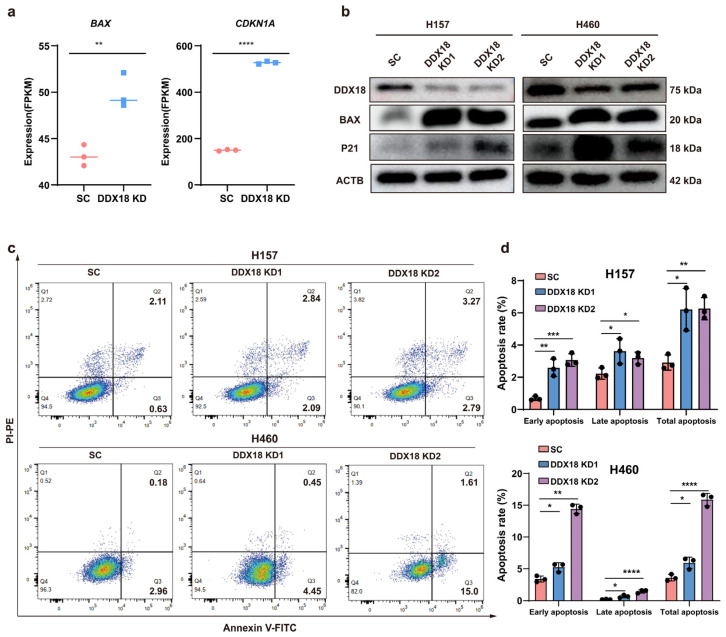
*DDX18* Knockdown Induces Apoptosis in LUAD Cells. (**a**) RNA-seq analysis reveals upregulation of *BAX* and *CDKN1A* mRNA levels following *DDX18* knockdown. (**b**) Western blot analysis confirms increased protein levels of BAX and P21 in H157 and H460 cells with *DDX18* knockdown. (**c**,**d**) Flow cytometry analysis of apoptosis induction in H460 and H157 cells with *DDX18* knockdown. Cells were stained with Annexin V (AV)/propidium iodide (PI) for double staining (**c**). Quantification of apoptotic cells (**c**) is shown in (**d**). * *p* < 0.05; ** *p* < 0.01; *** *p* < 0.001; **** *p* < 0.0001. SC, scramble control; KD, knockdown.

RNA-seq analysis revealed upregulation of apoptosis-related genes *CDKN1A* (P21) and *BAX* upon *DDX18* knockdown in H460 cells (Figure 7a). Western blot analysis confirmed these findings, demonstrating increased protein levels of P21 and BAX following *DDX18* knockdown (Figure 7b). To investigate whether apoptosis contributes to the growth inhibition observed in *DDX18*-depleted H460 and H157 cells, we performed flow cytometry analysis with Annexin V/PI double staining. This analysis revealed a significant increase in the total apoptotic cell population following *DDX18* knockdown (Figure 7c,d). Collectively, these results suggest that *DDX18* knockdown promotes apoptosis in LUAD cells.

### 2.7. DDX18 Regulates the Cell Cycle through Transcriptional Regulation of CDK4

**Figure 8:**
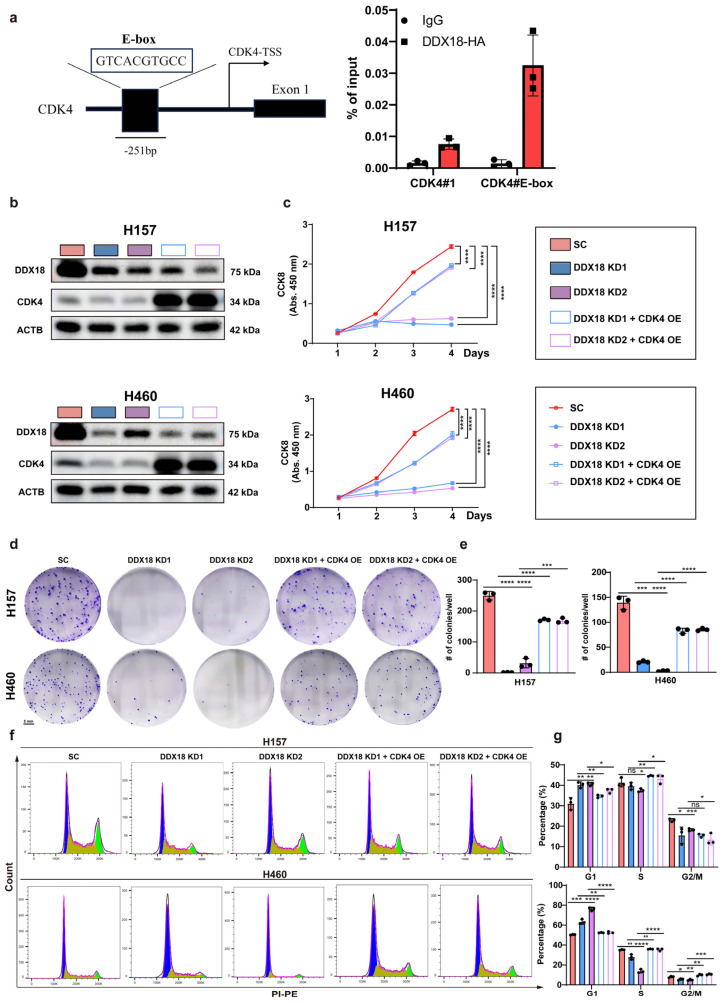
*DDX18* Regulates Cell Cycle via CDK4 Transcriptional Activation. (**a**) ChIP analysis demonstrates *DDX18* enrichment at the *CDK4* promoter region. (**b**) Western blot analysis confirms *DDX18* and CDK4 protein levels in H157 and H460 cells. (**c**–**e**) Rescue of CDK4 expression restores the proliferation capacity (**c**) and colony formation ability (**d**,**e**) of *DDX18* knockdown LUAD cells. (**f**) Flow cytometry analysis of cell cycle distribution in H460 and H157 cells with *DDX18* knockdown and CDK4 rescue. Cells were stained with propidium iodide (PI). Histograms depict cell counts (y-axis) versus DNA content (x-axis). (**g**) Quantification of cell cycle distribution data from panel F. Data represent the percentage of cells in each cell cycle phase (G1, S, G2/M). * *p* < 0.05; ** *p* < 0.01; *** *p* < 0.001; **** *p* < 0.0001; ns means non-significant. SC, scramble control; KD, knockdown; OE, overexpressed; CTL, control.

We identified a potential E-box element (GTCACGTGCC) located 251 bp upstream of the CDK4 transcriptional start site (TSS) (Figure 8a, left panel) based on the previous literature [33]. To investigate *DDX18* interaction with this E-box in H460 cells, we constructed an overexpression vector expressing HA-tagged *DDX18* and established H460 cells stably expressing this construct. Chromatin immunoprecipitation (ChIP) assay using an anti-HA antibody revealed enrichment of the *CDK4* promoter E-box region compared to a non-binding region 1798 bp upstream of the TSS (Figure 8a, right panel). These findings suggest that *DDX18* potentially binds to the *CDK4* promoter.

**Figure 9:**
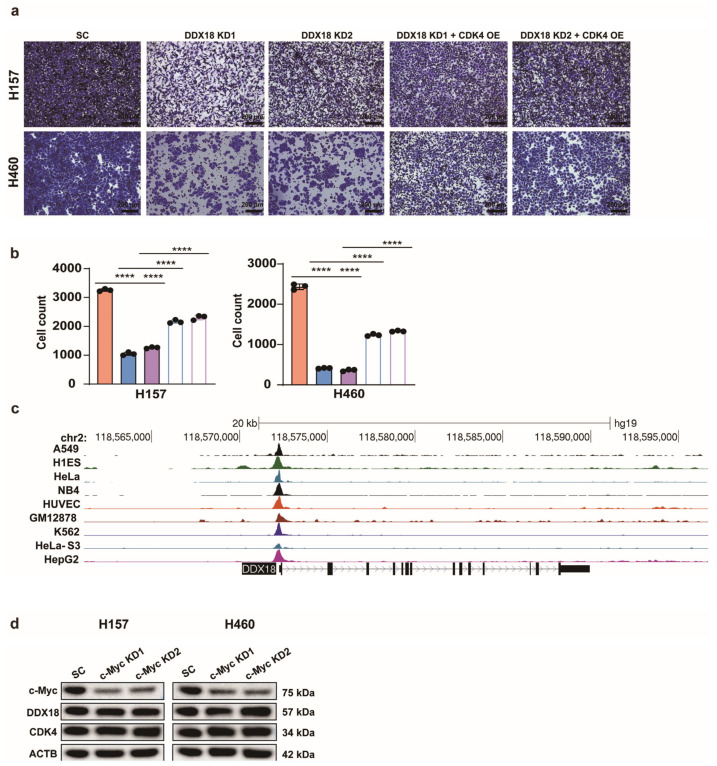
c-Myc Does Not Regulate *DDX18* Expression. (**a**,**b**) The migration of LUAD cells was restored by rescuing CDK4. The representative photos (**a**) and quantification of the migration cells in each condition (**b**) are shown. **** *p* < 0.0001. (**c**) Analysis of ChIP-seq data for c-Myc in ENCODE database cell lines predicted c-Myc binding to the *DDX18* promoter region. (**d**) Western blot analysis confirms that c-Myc knockdown does not affect the protein levels of *DDX18* or CDK4 in LUAD cells. SC, scramble control; KD, knockdown; OE, overexpressed; CTL, control.

To further establish CDK4 as a functional downstream target of *DDX18* in cell cycle regulation, we rescued CDK4 expression in *DDX18* knockdown LUAD cells. Western blot analysis confirmed restoration of CDK4 protein levels upon CDK4 rescue (Figure 8b). Notably, CDK4 rescue restored the proliferation capacity (Figure 8c) and colony formation ability (Figure 8d) of *DDX18*-depleted LUAD cells. Furthermore, CDK4 rescue significantly reduced the G1-arrested cell population as measured by flow cytometry (Figure 8f,g) and restored the migratory capacity of these cells (Figure 9a,b). Collectively, these data strongly suggest that CDK4 is a critical downstream effector of *DDX18*-mediated cell cycle regulation in LUAD.

### 2.8. c-Myc Does Not Regulate DDX18 Expression

c-Myc, a well-characterized oncogenic transcription factor known to regulate a vast array of genes, has been proposed to regulate *DDX18* expression through promoter binding [34]. To investigate this, we analyzed ChIP-seq data for c-Myc in publicly available ENCODE database cell lines. While the analysis predicted c-Myc binding to the *DDX18* promoter region (Figure 9c), c-Myc knockdown in LUAD cells did not affect *DDX18* protein levels (Figure 9d). These findings suggest that c-Myc may not be a functional regulator of *DDX18* expression in LUAD cells.

## 3. Discussion

While *DDX18* has been implicated in pancreatic and gastric cancers [30,31], its function in lung cancer and its contribution to lung cancer cell biology were unclear. In the present study, we demonstrate that *DDX18* expression is upregulated in lung cancer and correlates with poor prognosis. Furthermore, *DDX18* depletion significantly inhibits the proliferation, invasion, and migration of lung cancer cells in both in vitro and in vivo models. Mechanistically, we revealed that *DDX18* promotes lung cancer cell cycle progression through transcriptional activation of CDK4. Collectively, our findings unveil a novel oncogenic role for *DDX18* in lung cancer.

Limited research has previously explored the function of *DDX18*. Our findings regarding *DDX18*’s role in lung cancer progression enrich its multifaceted contributions in cancer biology. While previous work showed *DDX18*’s involvement in tumorigenesis across various cancers, the mechanisms appear to be highly context-dependent. In pancreatic cancer, *DDX18* promotes immune escape through STAT1 activation, shielding cancer cells from immune surveillance [30]. Conversely, in gastric cancer, *DDX18* fosters tumor development by impacting microRNA-21 maturation [31]. Interestingly, a separate study revealed a contrasting protective function for *DDX18*, where it interacts with PARP-1 to prevent R-loop formation and DNA damage, thereby safeguarding genome stability [32]. These seemingly disparate findings underscore the complex and context-dependent nature of *DDX18*’s influence on tumorigenesis. It is likely that *DDX18* exerts its effects through distinct regulatory pathways depending on the cellular context and specific tumor type. Further investigation into the precise mechanisms underlying *DDX18*’s context-specific functions in different cancers is warranted to elucidate its full oncogenic potential.

CDK4, a well-characterized cyclin-dependent kinase essential for G1/S phase transition, is frequently dysregulated in various cancers [35]. Studies have demonstrated that CDK4 deficiency impedes S phase entry, leading to cell cycle arrest in G1 [36]. Our findings provide compelling evidence that *DDX18* depletion induces G1 phase arrest in lung cancer cells, coinciding with a decrease in CDK4 protein expression. Conversely, *DDX18* overexpression accelerates cell cycle progression in normal lung cells. Mechanistically, we identified *DDX18* enrichment at the CDK4 promoter region, suggesting its role as a transcriptional activator of CDK4. This finding establishes a novel regulatory mechanism by which *DDX18* promotes cell cycle progression of lung adenocarcinoma through CDK4 upregulation. By targeting *DDX18* and its ability to regulate CDK4 expression and influence cell cycle progression, we can potentially develop novel therapeutic strategies to impede lung cancer growth.

c-Myc, a well-established oncogene frequently dysregulated in cancer [37], has been reported to bind the *DDX18* promoter [34], implying that it may potentially activate *DDX18* transcription. However, our findings suggest a disconnect between c-Myc expression and *DDX18* levels in lung cancer, highlighting the possibility of cell type-specific regulatory mechanisms or the existence of alternative upstream regulators of *DDX18*. This discrepancy underscores the need for further investigation into the upstream transcriptional control of *DDX18*. The main limitation of our present study is the lack of identification of the potential factor that regulates the transcription of *DDX18*. Identifying the key transcription factors involved in *DDX18*-CDK4 axis regulation is critical to fully elucidate the molecular mechanisms governing *DDX18* function in lung carcinogenesis.

Lung cancer development is a complex process driven by genetic alterations. Mutations in tumor suppressor genes, like TP53, and oncogenes, such as KRAS, play a key role in initiating uncontrolled proliferation of lung epithelial cells [38]. These mutations can be caused by exposure to carcinogens like tobacco smoke, which damages DNA and disrupts cell cycle regulation [38]. Additionally, chronic inflammation triggered by smoking or other factors can create a microenvironment promoting tumor growth and survival by releasing pro-inflammatory mediators and growth factors [39]. Furthermore, metabolic reprogramming is a hallmark of cancer, and lung tumors often exhibit increased glucose uptake and aerobic glycolysis, even in the presence of oxygen, to support their growth [40].

The expanding repertoire of RNA helicases implicated in tumorigenesis highlights their potential as therapeutic targets. Despite the lack of clinically validated drug targets within this family, ongoing research actively explores their therapeutic potential. Our findings on the significant role of *DDX18* in lung cancer progression warrant further investigation of its underlying molecular mechanisms. This will involve recruiting patients for clinical lung cancer sample collection and the development of primary mouse lung cancer models to dissect the impact of *DDX18* on the immune response. Our future endeavors will focus on the development or identification of potent and selective small-molecule inhibitors for *DDX18*. Employing these inhibitors for in vivo mouse lung cancer models will provide insights to inform the design of future clinical translation strategies.

## 4. Materials and Methods

### 4.1. Cell Culture

Human embryonic kidney 293 cells (HEK293 FT), bronchial epithelial cells (Beas-2B), and two non-small cell lung cancer cell lines (H460 and H157) were obtained from ATCC (American Type Culture Collection, Rockville, MD, USA). HEK293 FT and Beas-2b were cultured in Dulbecco’s Modified Eagle Medium (DMEM) containing 10% fetal bovine serum (FBS) and 1% penicillin/streptomycin. H460 and H157 cells were cultured in RPMI-1640 media (Invitrogen, Carlsbad, CA, USA) containing 10% FBS and 1% penicillin/streptomycin. All cell lines were incubated in a humidified chamber at 37 °C with 5% CO_2_.

### 4.2. Cell Viability Assay

The effects of *DDX18* depletion on the cell growth of three cell lines were measured by the Cell Counting Kit-8 (CCK-8) assay (Dojindo, Kumamoto, Japan). Cells were plated in 96-well plates at 3 × 10^3^ cells/well in 200 μL. After being cultured for 24, 48, 72, or 96 h, the medium was replaced with a mixture of 100 μL of the CCK-8 reagent diluted with RPMI-1640 (1:10). The plate was incubated for an additional 60 min, and then measured at 450 nm with a microplate reader. Independent experiments were repeated at least three times.

### 4.3. Colony Formation Assay

H460 and H157 cells were seeded onto 6-well plates with a density of 500 cells per well and cultured for about 1 week. Subsequently, the cells were fixed with methanol for 30 min and stained with crystal violet (Beyotime Biotechnology, Shanghai, China) for 30 min. Colonies were counted using ImageJ 1.8.0 software after washing and drying.

### 4.4. Cell Migration Assay

Culture medium containing 10% FBS (500 μL) was added to the wells of a 24-well plate, and 1 × 10^5^ cells were cultured with FBS-free medium in the inner cavity of the Transwell insert. After the incubation for 36 h, cells adhering to the Transwell insert membrane were fixed and were stained with crystal violet.

### 4.5. Cell Cycle Analysis

The cell cycle was quantified using the manufacturer’s instructions for the cell cycle analysis kit (Multi Science, Hangzhou, China). A single-cell suspension of H460 and H157 cells was prepared by trypsinization and washed once with phosphate-buffered saline (PBS). Subsequently, the cells were centrifuged (1000× *g* rpm, 5 min) and resuspended in 300 μL PI containing 3 μL immobilization in a dark place for 30 min. The samples were immediately analyzed by flow cytometry (Thermo Fisher Scientific, Waltham, MA, USA).

### 4.6. Apoptosis Assays

Apoptosis was quantified by using the Annexin V/propidium iodide (PI) kit (BD Biosciences, Franklin Lakes, NJ, USA). A single-cell suspension of H460 and H157 cells was prepared by trypsinization and washed once with PBS. After centrifugation (1000× *g* rpm, 5 min), cells were resuspended in 1× annexin V binding buffer 80 µL containing 1 µL Annexin V-FITC and 1 µL PI at 37 °C for 20 min. Cell apoptosis was evaluated by flow cytometry.

### 4.7. Real-time (RT) Quantitative PCR

Total RNA of cells and tumors was extracted through TRIZOL reagent (Invitrogen, Carlsbad, CA, USA), and 500 ng of the RNA was used with the reverse transcriptase (Accurate Biology, Hunan, China) to produce cDNA. Real-time qPCR was performed with an Applied Biosystems^TM^ QuantStudio^TM^ 3&5 (Thermo Fisher Scientific) using FastStart Universal SYBR Green Master (Takara, Otsu, Shiga, Japan). PCR conditions were the following: 3 min at 95 °C, 40 cycles of 10 s at 95 °C, and 30 s at 60 °C. The average Ct value for each gene was determined from triplicate reactions and normalized with mRNA levels of *GAPDH*. Specificity of the PCR products was confirmed by the presence of a single, well-defined melt peak following each PCR run.

Primer sequences were as follows:

*DDX18*-Forward: 5′-GTGGATGGTCTTGAACAGGGAT-3′

*DDX18*-Reverse: 5′-TCCATGAATGGCCAAGACGG-3′

GAPDH-Forward: 5′-TTCACCACCATGGAGAAGGC-3′

GAPDH-Reverse: 5′-GGCATGGACTGTGGTCATGA-3′

### 4.8. Chromatin Immunoprecipitation (CHIP)

Chromatin immunoprecipitation (ChIP) assays were performed using a SimpleChIP Enzymatic Chromatin IP kit (Cell Signaling Technologies, Danvers, MA, USA). Cells were fixed with 1% formaldehyde for 10 min, and glycine with 0.125 M was added to stop the fixation. Chromatin was treated with micrococcal nuclease, sonicated, and immunoprecipitated with normal rabbit IgG (negative control) or anti-HA antibody (Proteintech, Rosemont, IL, USA) overnight at 4 °C. After the reverse cross-linking and DNA purification, immunoprecipitated DNA was quantified by qPCR.

Primer sequences were as follows:

CDK4#1-Forward: 5′-GGACCCAAGCAGACAGAGAG-3′

CDK4#1-Reverse: 5′-GGTGGGTGCTTTGTAAGCCT-3′

CDK4#E-box-Forward: 5′-GTGGCCTAGGTTGCCATGGCAC-3′

CDK4#E-box-Reverse: 5′-CTCACCATGTGACCAGCTGCC-3′

### 4.9. Construct and Lentivirus Production

For *DDX18* knockdown, two different *DDX18* shRNA target sequences were synthesized and inserted into pLKO.1-puro vector (Plasmid #8453, Addgene, Watertown, MA, USA). To overexpress *DDX18*, full-length *DDX18* cDNA was inserted into pLenti-CMV GFP vector (Plasmid #17448, Addgene) linearized by BamHl/Sall digestion. For lentivirus production, package plasmid psPAX2 (Plasmid #12260, Addgene), envelop plasmid pMD2.G (Plasmid #12259, Addgene), and vectors were co-transfected into HEK 293FT cells. Then, 72 h after transfection, the supernatant was harvested and concentrated with Lenti-X concentrator (ECOTOP, Guangzhou, China). One day before the viral infection, cells were plated into a 6-well plate at a density of 1 × 10^5^ cells/well and transduced in the presence of 5 μg/mL polybrene for 24 h. The cells were selected for puromycin resistance (1.5 μg/mL) for 48 h.

### 4.10. Western Blotting

Western blotting was performed as previously described [23]. Membranes were probed with the following antibodies: *DDX18* (DF9258, Affinity Biosciences, Changzhou, China), CDK4 (AF2515, Beyotime, Shanghai, China), CDK6 (AG1575, Beyotime), CyclinD1 (ab32053, Abcam, Cambridge, MA, USA), BAX (ab32503, Abcam), P21 (ab109520, Abcam). All primary antibodies were diluted in Tris-buffered saline containing Tween 20 and 3% bovine serum albumin (BSA), and a peroxidase conjugate (Bioworld, Saint Louis Park, MN, USA) was used as the secondary antibody. GAPDH (60004-1-Ig, Proteintech, Rosemont, IL, USA) and β-ACTIN (20536-I, Proteintech) were used as a loading control. Immunoreactive bands were visualized using an enhanced chemo-luminescence kit (Thermo Fisher Scientific), and images were captured using a Amersham^TM^ ImageQuant^TM^ 800 molecular imager (Cytiva, Washington, DC, USA).

### 4.11. Establishment of the Xenograft Mouse Model

All work with the xenograft mouse model was conducted following the guidelines of the Ministry of Science and Technology of China and was approved by the Animal Ethics Committee of Sun Yat-sen University. Six-week-old NU/NU mice were obtained from the Guangdong Experimental Animal Center. Animals were maintained under a 12 h light/dark cycle in a specific pathogen-free (SPF) facility with ad libitum access to standard laboratory chow and water. To assess the tumor growth, 5 × 10^6^ H460 cells were injected subcutaneously into the left (control group) and right (*DDX18* knockdown group) flanks of each mouse. Tumor sizes were measured with a caliper and recorded every day. Tumor volume was calculated as follows: tumor volume = (length × width^2^) × 0.5, and when the size reached ~1.5 cm^3^, mice were euthanized, and tumor weight was measured after removing the tumors.

### 4.12. RNA Sequencing and Bioinformatics Analysis

RNA isolation for RNA sequencing (RNA-seq) analyses was performed using TRIZOL reagent (Invitrogen). The whole transcriptome libraries for costunolide and DMSO-treated cells (n = 3) were prepared with the SOLiD TM Whole Transcriptome Analysis Kit (Applied Biosystems, Carlsbad, CA, USA) and paired-end sequencing was conducted on an Illumina HiSeq system using PE150 strategy. The reads were aligned against the human reference genome (H. sapiens, GRCh38) and TMM normalization method was used for data normalization (R/Bioconductor package edgeR). Limma package and Student’s *t*-test were used for statistical analysis of the mean expression values between costunolide and DMSO-treated lung cancer cell lines (n = 3). KEGG and Reactome analysis were conducted by KOBAS3.0 (kobas.cbi.pku.edu.cn/kobas3 (accessed on 13 July 2023)) [41], and pathways with *p*-value < 0.05 were considered to be significantly enriched in the annotation categories.

### 4.13. Statistical Analysis

All experimental data in the figures are shown as the mean ± SD. The statistical significance of Kaplan–Meier survival plot was determined by log-rank analysis. The other statistical significance was detected by *t*-test. All of the statistical analyses were performed in GraphPad Prism 8.3.0. * *p* < 0.05, ** *p* < 0.01, *** *p* < 0.001, and **** *p* < 0.0001; ns means non-significant. All experiments were repeated at least three times.

## 5. Conclusions

Our findings demonstrate a potential oncogenic role for *DDX18* in LUAD. Analysis of TCGA and GEO databases revealed significant upregulation of *DDX18* in LUAD tissues, and this upregulation correlated with poor patient survival. Functional studies confirmed that *DDX18* knockdown in LUAD cells impedes cell growth, while overexpression in Beas-2B cells promotes proliferation. RNA-seq identified the cell cycle pathway as significantly dysregulated upon *DDX18* depletion. Flow cytometry analysis further revealed G1 cell cycle arrest in *DDX18* knockdown cells, accompanied by downregulation of the key G1 regulator CDK4. CHIP experiments confirmed *DDX18* binding to the CDK4 promoter region. Rescue of CDK4 expression after *DDX18* knockdown abrogated the cell cycle arrest phenotype, suggesting *DDX18* regulates cell cycle progression via CDK4 expression. Collectively, these data strongly support the potential of *DDX18* as a therapeutic target for lung cancer.

## Data Availability

The RNA-seq data supporting the conclusion of this article are available in the Gene Expression Omnibus Repository (GSE75037). The data from in vivo and in vitro experiments in the article are available by contacting the corresponding author.

## Abbreviations

LUAD	Lung adenocarcinoma
*DDX18*	DEAD-box RNA helicase 18
CCK8	Cell Counting Kit-8
TCGA	The Cancer Genome Atlas Program
GEO	Gene Expression Omnibus
CDK	cyclin-dependent kinase
GSEA	Gene Set Enrichment Analysis
DMSO	Dimethyl sulfoxide
RNA-seq	RNA sequencing
CHIP	Chromatin immunoprecipitation
FBS	fetal bovine serum
SCLC	small cell carcinoma
NSCLC	non-small cell carcinoma
RB	retinoblastoma protein
YB1	Y-box binding protein 1
EGFR	epidermal growth factor receptor
PI	propidium iodide
H&E	Hematoxylin and eosin
TSS	transcriptional start site
KEGG	Kyoto Encyclopedia of Genes and Genomes
GO	Gene Ontology
DEGs	differentially expressed genes
